# Retraction: Up-Regulation of pVHL along with Down-Regulation of HIF-1α by NDRG2 Expression Attenuates Proliferation and Invasion in Renal Cancer Cells

**DOI:** 10.1371/journal.pone.0095904

**Published:** 2014-04-16

**Authors:** Lei Gao, Guo-jun Wu, Bei Liu, Ming-zhi Shen, Tie-jun Pan, Chui-gong Yu, Qin-hao Wang, Yi Ru, Xi-ping Liu, Tian-shui Niu, Guo-dong Wang, Ming Wei, Rui-xiao Li, Libo Yao, He Wang, Xia Li

The authors retract this publication due to concerns about the cell lines employed in the study.

One part of the study involves an assessment of the effects of NDRG2 on the expression of pVHL, HIF-1α and VEGF in 786-O cells. After the publication of the article, a reader raised concerns that the human renal cancer cell lines 786-O and A498 used in the study lack HIF-1α. In addition the cell line 786-O has been reported to be VHL negative. The authors have completed a short tandem repeat analysis on the cell lines and this has revealed cross-contamination, which compromises the relevance of the work to renal cell carcinoma, and thus the conclusions of the study.

In the light of this, the authors retract this publication.
